# Cellular Model of Atherogenesis Based on Pluripotent Vascular Wall Pericytes

**DOI:** 10.1155/2016/7321404

**Published:** 2016-01-05

**Authors:** Ekaterina A. Ivanova, Alexander N. Orekhov

**Affiliations:** ^1^Department of Development and Regeneration, KU Leuven, 3000 Leuven, Belgium; ^2^Laboratory of Angiopathology, Institute of General Pathology and Pathophysiology, Moscow 125315, Russia; ^3^Institute for Atherosclerosis Research, Skolkovo Innovative Center, Moscow 121609, Russia

## Abstract

Pericytes are pluripotent cells that can be found in the vascular wall of both microvessels and large arteries and veins. They have distinct morphology with long branching processes and form numerous contacts with each other and with endothelial cells, organizing the vascular wall cells into a three-dimensional network. Accumulating evidence demonstrates that pericytes may play a key role in the pathogenesis of vascular disorders, including atherosclerosis. Macrovascular pericytes are able to accumulate lipids and contribute to growth and vascularization of the atherosclerotic plaque. Moreover, they participate in the local inflammatory process and thrombosis, which can lead to fatal consequences. At the same time, pericytes can represent a useful model for studying the atherosclerotic process and for the development of novel therapeutic approaches. In particular, they are suitable for testing various substances' potential for decreasing lipid accumulation induced by the incubation of cells with atherogenic low-density lipoprotein. In this review we will discuss the application of cellular models for studying atherosclerosis and provide several examples of successful application of these models to drug research.

## 1. Introduction

Mural cells isolated from the human vascular wall represent a heterogeneous population, containing pericytes or pericyte-like cells together with other cell types. Pericytes, or perivascular cells, are characterized by branched morphology and the formation of numerous contacts between each other and with endothelial cells (ECs). Pericytes are embedded into the basement membrane of the vascular wall and wrap around the ECs [1, 2]. They are responsible for microvascular growth and branching [3]. Pericytes can be found in* vasa vasorum* microvessels that provide alimentation of wall tissue of large blood vessels [4, 5]. Furthermore, presence of stellate pericyte-like cells in the intima of large arteries and veins has been reported in a series of studies [6, 7].

Pericytes are pluripotent cells and can differentiate to other cell types, such as myofibroblasts, osteoblasts, and adipocytes. It is possible that pericytes possess regenerative properties in healthy tissue participating in vascular remodelling and vascular injury repair [8]. At the same time, pericytes may play a central role in the development of vascular pathologies. The discovery of pericytes and pericyte-like cells in the large arteries highlights the possibility of their involvement in the atherosclerotic process. Indeed, recent studies demonstrated that pericytes play important roles in atherogenesis, accumulating lipids, promoting the atherosclerotic plaque growth and vascularization, participating in vascular remodelling, calcification, and thrombosis (reviewed in [6]). It is also likely that these pluripotent cells may express proinflammatory molecules and thus orchestrate the local inflammation, which plays an important role in atherosclerosis. Pericytes and pericyte-like cells can be extracted from postmortem samples of human aortic tissue and used for studying atherogenesis, as well as testing various substances with potential antiatherosclerotic activity. Such cellular models are highly relevant and therefore very promising for atherosclerosis research and drug development.

## 2. Cellular Structure of Human Arterial Intima

**Figure 1:**
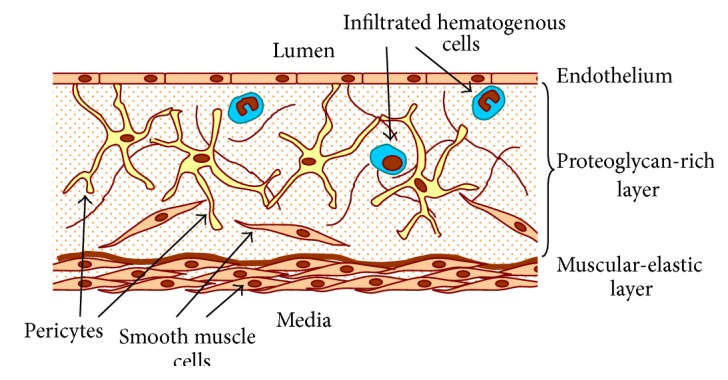
Schematic representation of the proteoglycan-rich layer of human aortic intima. Stellate macrovascular pericytes form a three-dimensional cellular network in the subendothelial layer of intima forming contacts with each other and other cell types.

The human arterial wall consists of several distinct layers (Figure 1). Immediately below the endothelial lining of the artery lies the proteoglycan-rich layer, which is also known as elastic-hyperelastic or connective-tissue layer. It contains connective-tissue fibres that have no distinct orientation and a heterogeneous cell population. Proteoglycan-rich layer is separated from more distal layers of the arterial wall by the internal limiting membrane. Beyond the internal limiting membrane lies the muscular-elastic layer, which consists of longitudinally oriented elongated cells and elastic fibres. The layers differ from each other both by the cell population and by the composition of glycosaminoglycans [6].

Cellular composition of the subendothelial proteoglycan-rich layer is heterogeneous, as the layer contains both resident cells and monocytes/macrophages and lymphocytes that infiltrate into the intima from the bloodstream [9]. However, in healthy human aortic intima, cells of haematogenous origin represent the minority, accounting for as little as 5% of the total cell population [6]. Among the resident cells, vascular smooth muscle cells, characterized by *α*-smooth muscle actin (*α*SMA) expression, are the best studied population. In contrast to the more distal muscular-elastic layer, there, these cells are arranged in regular sheets separated by elastic fibres and proteoglycan-rich layer contains randomly oriented smooth muscle cells that form contacts with each other.

The most intriguing type of the intimal cells is stellate, or pericyte-like, cells that are characterized by pluripotency and by distinct morphology with long processes contacting other cells. Their identity has long remained uncertain and different explanations of their origin have been suggested. Some researchers proposed that these cells represent a modified phenotype of vascular smooth muscle cells [10, 11]. However, early electron microscopy study revealed some ultrastructural features linking them to the pluripotent mesenchymal progenitor cells [12]. Alternatively, it has been proposed that the intimal stellate cells represent the pluripotent pericytes or pericyte-like cells [6, 7, 13].

## 3. Pericyte Phenotype and Plasticity

Identification of pericytes is complicated because of the flexible phenotype of these cells. The expression of most of the proteins that can be used as pericyte markers is dynamic and depends on anatomical location of the cells. Many of these proteins are also shared between pericytes and vascular smooth muscle cells, hindering the accurate identification [14]. *α*-smooth muscle actin (*α*SMA) is expressed by pericytes located in contractile capillaries depending on the vasoconstriction activity and/or the maturity of pericytes [15, 16]. Apart from *α*SMA, commonly used markers for pericyte identification include CD146, PDGFR-*β*, regulator of G protein signalling 5 (RGS-5), and neuron-glial 2 (NG2) [17, 18]. An extended list of markers also includes aminopeptidases A and N (CD13), endoglin, nonmuscle myosin, desmin, vimentin, and nestin [14, 19]. However, most of these markers are shared with other cell types. For instance, CD146 is also expressed on vascular endothelial cells and PDGFR-*β* on fibroblasts and astrocytes [20]. Therefore, the identification of pericytes is best performed using a combination of known markers. For example, an immunocytochemical analysis of pericytes could be performed using a combination of *α*SMA and NG2 expression and negative expression of endothelial cell (CD34 and CD31) and leukocyte markers [21].

The majority of the resident intimal cells were found to be *α*SMA-positive, especially in the muscular-elastic layer. The percentage of *α*SMA-positive cells in the proteoglycan-rich layer is much lower, and these cells have different morphology than the typical smooth muscle cells, having long processes and being less densely packed. Interestingly, some of the *α*SMA-positive cells in the proteoglycan-rich layer expressed markers, unusual for smooth muscle cells, such as CD68 [6, 22]. Moreover, it has been demonstrated that some antigens expressed on resident intimal cells were not present on the vascular smooth muscle cells. Antigen 3G5 is an O-sialoganglioside, which was found to be specific for microvascular pericytes [7]. It was detected on a subset of resident intimal cells but not on vascular smooth muscular cells [23]. In human aortic tissue, cells positive for 3G5 were found to have typical pericyte morphology, forming a network with their long processes, and accounted for approximately one-third of total intimal cells. Macrovascular intima also contained cells positive for 2A7 antigen, a melanoma chondroitin sulphate proteoglycan, which is typical for activated pericytes [24]. The expression of these markers was demonstrated to be altered in association with atherogenesis: incubation of primary subendothelial cells with atherogenic modified LDL resulted in the reduction of 3G5 expression and the increase of 2A7 expression [24]. The 2A7 antigen was suggested to be expressed on activated pericytes, whereas 3G5 was suggested to be expressed on quiescent pericytes [25].

Taken together, these observations strongly suggest that pericytes are present in the subendothelial layer of human macrovascular intima, where they maintain a cellular network and form contacts with the endothelial cells.

## 4. Subendothelial Intima and Pericytes in the Pathogenesis of Atherosclerosis

The role of pericytes and pericyte-like cells in the disease pathogenesis is currently being widely recognized. Pericytes appear to be involved in various conditions associated with impaired microcirculation, such as diabetes, inflammation, wound healing, and tumor growth. Converging evidence demonstrates that macrovascular pericytes may play an important role in atherosclerosis [6].

Pericytes are pluripotent cells capable of differentiation into other cell types in the vascular wall. Such plasticity may be important for maintaining the normal structure of the vascular wall and the repair of injuries but can also be implicated in pathological processes, such as atherosclerosis. It has been demonstrated that pericytes can perform active phagocytosis and express the macrophage-specific CD68 antigen [22]. This feature is especially relevant regarding lipid accumulation in the atherosclerotic process, which leads to the formation of foam cells and thickening of the arterial wall. Like fibroblasts, pericytes can proliferate and participate in the extracellular matrix synthesis, also contributing to the arterial wall thickening in atherosclerosis. Moreover, there are indications that pericytes, together with dendritic cells, participate in antigen presentation in the vascular wall, as microscopic studies have revealed a network of stellate interconnected subendothelial cells expressing the major histocompatibility complex class II (MHC II) molecule HLA-DR [6, 26].

Atherosclerotic plaque progression consists of several stages: from unaffected intima to the initial lesions, fatty streaks, lipofibrous plaques, and, finally, fibrous plaques. Comparative studies of grossly normal aortic intima and atherosclerotic plaque regions demonstrated that thickening of the proteoglycan-rich layer in the affected regions was accompanied by the increased cell count and enhanced proliferation of intimal cells. The maximal number of cells was detected in lipofibrous plaques. Both infiltration of immune cells and increased proliferation of resident intimal cells accounted for the increased cellularity, although the input of the increased proliferation varied depending on the plaque location and the progression stage [6, 27].

Because of their subendothelial location, macrovascular pericytes are sensitive to the atherogenic stimuli, such as presence of atherogenic modified LDL and proinflammatory signals, and can play an important role already at the early stages of lesion development. These cells can actively accumulate lipids, which can lead to their activation and proliferation, as well as to differentiation to other cell types contributing to the plaque growth and vascular calcification [23]. Lipid accumulation by pericytes leads to the increase of cell size and loss of cellular Connexin 43- (Cx43-) mediated contacts and therefore disturbance of the subendothelial cellular network [28]. The LDL-induced phenotypic changes observed in atherosclerosis are accompanied by upregulated expression of T-cadherin, an unusual member of the cadherin family of adhesion molecules [29]. This observation highlights the possibility of disturbed signalling processes mediated by T-cadherin, such as activation of Erk1/2 tyrosine kinase and NF-*κ*B nuclear translocation [30]. T-cadherin can also mediate the LDL-induced chondrogenic differentiation of pericytes and vascular wall remodelling in atherosclerosis through Wnt/*β*-catenin pathway. Activation of this pathway blocks the adipogenic and promotes the chondrogenic differentiation of pericytes associated with increased glycosaminoglycan accumulation in the extracellular matrix [31]. The enhanced transforming growth factor beta (TGF-*β*) signalling in the atherosclerotic lesion can further promote this process [32].

At later stages, the atherosclerotic plaque develops a fibrous cap, which separates it from the bloodstream. Although growing plaque causes a significant narrowing of the artery lumen and can by itself cause ischemia of the alimented organs, the most dangerous are so-called vulnerable plaques with unstable fibrous cap that become sites of thrombogenesis. Thrombus formation followed by thromboembolic occlusion of vital arteries accounts for the large part of fatal events associated with atherosclerosis. Macrovascular pericytes may play a key role in this process being the source of thrombogenic tissue factor [7]. Overexpression of tissue factor promotes the aggregation of platelets to the endothelium-denuded arterial wall in the advanced-stage atherosclerotic plaque.

It can be therefore concluded that pericytes play a central role in the development of atherosclerotic lesions at the cellular level, participating in proliferation, intracellular lipid accumulation, and extracellular matrix synthesis. Microvascular pericytes of* vasa vasorum* can also contribute to the atherosclerotic plaque growth by orchestrating neovascularization of growing plaques [6].

## 5. Cellular Models Based on Primary Culture of Subendothelial Cells

Current therapy of atherosclerosis is largely based on the use of lipid-lowering drugs, such as statins [33, 34]. However, no therapy with direct antiatherosclerotic activity has been developed so far. Screening for therapeutic agents allowing the reduction of lipid accumulation in the arterial wall and atherosclerotic plaque growth requires development of adequate disease models. Given that subendothelial cells are playing a prominent role at all stages of the pathologic process including the initial lesion formation, primary culture of these cells appears to be promising for establishing such models. Cellular tests based on these cultures can be used for studying the early stages of atherogenesis and blood serum atherogenicity. Such tests are readily available and suitable for testing of large panels of substances prior to preclinical and clinical studies. Moreover, they are based on cell types that directly participate in lipid accumulation in atherosclerosis [35]. Here we describe two types of such models that have been successfully used for screening of different substances for potential antiatherosclerotic activities.

*In vitro* model of early stages of atherogenesis was based on primary culture of human aortic cells isolated from the subendothelial part of normal (unaffected) aortic intima [36]. Treatment of the autopsy tissue samples with collagenase and elastase resulted in obtaining heterogeneous population of living cells, including smooth muscle cells (20–50%), pericyte-like cells (30–70%), and inflammatory hematogenous cells and tissue macrophages (10%) [36]. Smooth muscle cells and stellate-shaped pericyte-like cells were *α*SMA-positive and represented the majority of the cell population. Incubation of this cell population with atherogenic serum obtained from patients with confirmed atherosclerosis resulted in cellular lipidosis, which could be measured by biochemical methods. To mimic the conditions of the increased risk of atherosclerosis, cells were incubated with serum obtained from atherosclerotic patients, which contains modified LDL particles and, probably, numerous other factors inducing lipid accumulation and initiation of atherosclerotic process in arterial wall cells. The ability of the serum to induce lipid accumulation in cultured cells is referred to as serum atherogenicity, and it may vary between individuals and upon treatment of patients with antiatherosclerotic medications. Atherogenic serum induced a twofold increase on intracellular cholesterol after 24 hours of incubation. For studying the antiatherogenic potential, tested substances were added to the cell culture together with the atherogenic serum, and the effect was measured as inhibition of lipid accumulation [36].

*Ex vivo* model made use of blood samples from subjects with sufficient blood serum atherogenicity before and after administration of tested drugs or natural products. Changes of the atherogenic properties of the blood serum were evaluated on the primary culture deriving from unaffected human aortic intima [36, 37]. The antiatherogenic activity of the tested substance was evaluated by the decrease of blood serum atherogenicity (the ability to induce intracellular lipid accumulation in cultured arterial wall cells). For evaluation of short-term antiatherosclerotic effects, blood samples were collected 2, 4, and 6 hours after the substance administration. Long-term effects were evaluated after 4, 8, 12, and 24 hours. This experimental setup allows evaluation of potential antiatherosclerotic substances taking into account their metabolic processing in patient's organism [36].

## 6. Application of Cellular Models to Drug Development

The described cellular models have been successfully used for screening natural substances of botanical origin for antiatherosclerotic activity. Natural substances are advantageous for prevention and treatment of early stages of atherosclerosis because of their good tolerability and suitability for long-term and even life-long therapy that are necessary for subjects with increased risk of cardiovascular diseases. Although many botanicals are widely used in traditional medicine, their efficacy has to be proven in accurate laboratory and clinical studies in order to develop therapeutic products.

Several of the substances tested using the cellular models were proven to be effective to reduce cellular lipid accumulation and blood serum atherogenicity [36]. The effect of botanicals on blood serum atherogenicity was studied on the* ex vivo* model. Onion bulb in a form of encapsulated powder provided a mild antiatherogenic effect, decreasing blood serum atherogenicity by 12%, 28%, and 24% from baseline 2, 4, and 6 hours after administration to the study subjects, respectively [36]. Most pronounced antiatherosclerotic activity was demonstrated for wheat seedlings and garlic powder (in capsules). Administration of garlic powder resulted in a reduction of blood serum atherogenicity 3-fold after 6 hours [36]. The beneficial properties of garlic have been studied for a long time, and its ability to reduce blood cholesterol level, lower the arterial pressure, and increase the fibrinolytic activity of blood plasma has been demonstrated by several groups [38–41]. Studies on cellular models clearly demonstrated the direct antiatherosclerotic activity of garlic powder making it an attractive substance for development of therapeutic products. To further study the antiatherosclerotic properties of garlic compounds, chloroform-soluble and water-soluble fractions were prepared using thin layer chromatography and tested in* in vitro* model based on aortic cells primary culture. Most of the chloroform-soluble fractions possessed both antiatherosclerotic and antiatherogenic activity, whereas water-soluble fractions displayed mostly antiatherogenic activity. These studies resulted in the development of garlic-based nonpharmaceutical commercial product Allicor (INAT-Pharma, Russia). The product is intended to be used for prevention and treatment of early stages of atherosclerosis and is suitable for long-term therapy. The antiatherosclerotic efficacy of the product was later confirmed in phases I–III clinical trials [36].

Inflammation plays a prominent role in the pathogenesis of atherosclerosis at all stages [42, 43]. The most important interleukins mediating the inflammatory process in atherosclerotic lesion are IL-1 and IL-6, with IL-1 inducing the local inflammation and IL-6 acting as a proinflammatory factor [44]. During the subclinical period of atherosclerosis development, the elevated level of inflammation markers can be detected in the blood serum, which correlates with the risk of atherosclerosis complications. It would be therefore interesting to develop anticytokine therapy suitable for long-term treatment of early stages of atherosclerosis. Using the described* in vitro* model, 31 botanical substances were tested for anticytokine activity. Five of them (violet, calendula, elder, hawthorn, and St. John's wort) demonstrated the ability to decrease the IL-1 expression and were selected for future research. The anticytokine activity of the botanicals was tested in the* ex vivo* model. Study subjects received a single dose of botanicals combination, and blood samples were drawn before and 4 and 8 hours after the administration. Violet, calendula, and elder were proven to be the most potent in reducing the expression of IL-1 and tumor necrosis factor alpha (TNF-*α*), and the combined product was developed on the basis of these botanicals in a form of capsules (Inflaminat, INAT-Pharma, Russia). The efficacy of the new product was tested in comparison with Diclofenac and Allicor on subjects with detected blood serum atherogenicity and elevated proinflammatory profile. Blood samples were drawn before and 2, 4, and 8 hours after the single dose administration and evaluated using the described* ex vivo* model. Administration of Inflaminat resulted in average decrease of IL-1 expression by almost 25%, while the control Diclofenac reduced the IL-1 expression by 49%. Comparable results were obtained for TNF-*α* expression reduction (9% reduction after Inflaminat and 39% after Diclofenac administration after 8 hours). Atherogenic potential of the blood serum was decreased after Inflaminat administration by 64%, after Allicor administration by 50%, and after Diclofenac administration by 13% after 8 hours. Therefore, the combined botanical product Inflaminat demonstrated anti-cytokine and antiatherosclerotic activity and was recommended for future clinical trials [45]. The effect of Inflaminat on atherosclerosis development was evaluated in a double-blind placebo-controlled study (ClinicalTrials.gov Identifier, NCT01743404), using carotid intima media thickness (cIMT) as readout. The product was demonstrated to reduce cIMT in subclinical atherosclerosis in comparison to the baseline and the placebo group [46].

Postmenopausal women represent a special group at risk of atherosclerosis development. Atherosclerosis-related disorders are the primary causes of sudden death in women (up to 73%). Hormone replacement therapy is effective against the menopausal symptoms but does not significantly reduce the risk of atherosclerosis development [47]. Phytoestrogens are currently regarded as a nonaggressive alternative for reducing the climacteric syndromes suitable for long-term therapy that have also potential for reducing the risk of cardiovascular disorders [48, 49]. The described* in vitro* and* ex vivo* models based on the culture of human aortic cells were used for evaluation of possible beneficial effects of phytoestrogen-containing compounds on the prevention of the atherosclerosis development. A panel of botanical was tested in the* ex vivo* model for the possible antiatherogenic activity, including grape, soybean, sage, carrot, orange, garlic, liquorice, onion, hop, green tea,* Fucus*, kelp, calendula, clover, hawthorn, elder, and violet. As a result, the most promising compounds, including tannin from grapes, garlic, hop, sage, and green tea leave, were selected for further studies. Study of dose-dependent antiatherogenic activity of these compounds was performed on the* ex vivo* model, and the final proportion of active compounds was generated in accordance with minimal effective dose of each substance and proposed scheme of daily treatment. As a result of these studies, a novel nonpharmaceutical phytoestrogen complex Karinat (INAT-Pharma, Russia) was developed. The product was recommended for future clinical studies to confirm its antiatherosclerotic activity [50]. To evaluate the antiatherosclerotic effect of Karinat, a randomized double-blind placebo-controlled study was performed in 157 asymptomatic postmenopausal women (ClinicalTrials.gov Identifier, NCT01742000). The effect of the product was assessed by the annual rate of cIMT. The study demonstrated that treatment with Karinat allowed stabilization of cIMT progression (mean rate < 1% per year) in comparison with the control group (mean rate 13% per year). Therefore, the phytoestrogen complex was proven to be efficient against the atherosclerotic lesion progression in this group of patients [50, 51].

## 7. Conclusion

Arterial wall cells play important roles in the development of vascular pathologies, including atherosclerosis. Among them, pericytes appear to be most interesting because of their pluripotency and potential direct contribution to such processes and thrombogenesis, vascular remodelling and calcification, inflammation, and atherosclerosis plaque growth. This makes pericytes an interesting model for studying the mechanisms of vascular disorders and for drug development. Primary culture of arterial wall cells contains a heterogeneous cell population that includes pericytes and vascular smooth muscle cells. As these resident arterial wall cell types are capable of active lipid accumulation, they can be used as models for studying blood serum atherogenicity and screening for substances reducing the blood atherogenicity and lipid accumulation in the arterial wall. Lipid accumulation in these cells can be induced by incubation with atherogenic modified LDL or atherogenic blood serum. The effect of the potential active substances can be evaluated as a decrease of lipid accumulation by cultured cells treated with the substance (*in vitro* model) or as decrease of blood serum atherogenicity after administration of the substance (with confirmed good safety profile) to study subjects (*ex vivo* model). Both models have been successfully used for development of several botanical-based nonpharmaceutical products intended for long-term treatment of atherosclerosis and preventive treatment of subjects at risk of atherosclerosis development. The described models can be useful for future drug research in the atherosclerosis field.
